# Immunometabolic reprogramming and mitochondrial dysfunction in acute respiratory distress syndrome: mechanisms, metabolic resilience, and therapeutic perspectives- a narrative review

**DOI:** 10.1186/s12967-026-08585-1

**Published:** 2026-07-10

**Authors:** Thejesh Srinivas, Gagana Hanumaiah, Danavath Nagendra, Shwethapriya R., Souvik Chaudhuri, Vinutha R. Bhat, Shobha U. Kamath

**Affiliations:** 1 Department of Critical Care Medicine, Kasturba Medical College, Manipal Academy of Higher Education, Manipal, Karnataka India; 2 Department of Obstetrics and Gynaecology, Kasturba Medical College, Manipal Academy of Higher Education, Manipal, Karnataka India; 3 Department of Biochemistry, Kasturba Medical College, Manipal Academy of Higher Education, Manipal, Karnataka India

**Keywords:** Acute respiratory distress syndrome (ARDS), Immunometabolism, Mitochondrial dysfunction, Metabolic resilience, Clinical trajectories

## Abstract

**Background:**

Acute respiratory distress syndrome (ARDS) is a biologically heterogeneous condition in which patients exposed to similar injurious stimuli often develop markedly different clinical trajectories and outcomes. While inflammation is central to ARDS pathogenesis, inflammatory burden alone does not fully explain the variability in disease progression, treatment response, or recovery. Emerging evidence suggests that immunometabolic reprogramming, characterized by increased glycolysis, impaired mitochondrial oxidative phosphorylation, and altered metabolite signalling, plays a critical role in shaping immune-cell activation, inflammatory persistence, and tissue repair during critical illness.

**Main body:**

This narrative review synthesizes current evidence linking immunometabolic reprogramming and mitochondrial dysfunction to the clinical heterogeneity observed in ARDS. During acute lung injury, immune and structural lung cells undergo metabolic shifts characterized by increased glycolysis, impaired mitochondrial oxidative phosphorylation, and accumulation of bioactive metabolites such as lactate, succinate, and extracellular adenosine triphosphate (ATP). Beyond reflecting metabolic stress, these metabolites function as signalling mediators that are associated with amplified inflammatory pathways, compromised alveolar-capillary barrier integrity, and sustained lung injury. Multi-omic studies further demonstrate that distinct metabolic signatures are associated with ARDS phenotypes, disease severity, and treatment responsiveness. We integrate these findings within the concept of metabolic resilience, defined as the host’s capacity to restore coordinated mitochondrial function, redox balance, and substrate utilization following inflammatory stress. Therapeutic strategies aimed at preserving mitochondrial function, restoring nicotinamide adenine dinucleotide (NAD⁺) homeostasis, and modulating maladaptive immunometabolic signalling may offer new avenues for precision-based interventions in ARDS.

**Conclusions:**

Immunometabolic reprogramming and mitochondrial dysfunction represent central biological axes linking cellular bioenergetics with clinical heterogeneity in ARDS. Understanding metabolic resilience may help refine phenotyping strategies and support development of metabolism-targeted therapies aimed at improving outcomes in this complex syndrome.

**Clinical trial number:**

Not applicable.

## Introduction

Acute respiratory distress syndrome (ARDS) is defined by the acute onset of hypoxemia and bilateral radiographic opacities not fully attributable to cardiac failure, reflecting a broad clinical phenotype rather than a single pathological entity. ARDS is characterized by diffuse alveolar damage, increased alveolar-capillary permeability, and severe hypoxemic respiratory failure. Despite advances in lung-protective ventilation and conservative fluid management, mortality remains high, and survivors often undergo prolonged respiratory and functional impairment. A major unresolved challenge in ARDS is its marked biological and clinical heterogeneity, where patients exposed to similar precipitating insults frequently follow divergent trajectories, with some recovering rapidly while others progress to refractory hypoxemia and multiorgan failure [1, 2].

Historically, ARDS pathogenesis has been primarily conceptualized through an inflammation-centric model, in which excessive cytokine release and immune activation drive lung injury. However, numerous clinical trials targeting individual inflammatory markers have failed to demonstrate consistent survival benefit, showing that inflammatory burden alone cannot fully explain disease progression, variability in treatment response, or ultimate outcomes. These observations suggest that upstream biological processes modulating host response to injury remain incompletely understood [3, 4].

Emerging data from systems biology and multi-omic studies indicate that cellular metabolism is tightly integrated with immune activation, epithelial repair, and endothelial barrier stability [5]. Rather than serving merely as an energy supply system, cellular metabolism functions as an active regulatory network that governs immune cell phenotype, redox balance, and tissue resilience during critical illness. Disruptions in bioenergetic homeostasis, including impaired mitochondrial oxidative phosphorylation, altered glycolytic flux, defective fatty acid oxidation, and redox imbalance have increasingly been linked to persistent organ dysfunction and adverse outcomes in ARDS and sepsis [6–8].

Clinical phenotyping studies have identified a hyperinflammatory ARDS subphenotype characterized by markedly elevated inflammatory biomarkers, greater vasopressor use, metabolic acidosis, fewer ventilator-free days, and substantially higher mortality compared with hypo inflammatory phenotypes [9]. Emerging clinical evidence from human cohorts supports the presence of metabolic dysregulation in sepsis-induced acute lung injury (ALI)/ARDS. Quantitative plasma proton nuclear magnetic resonance (^1^H-NMR) metabolomic profiling demonstrated significant alterations in metabolites associated with oxidant stress, energy homeostasis, apoptosis, and endothelial barrier dysfunction when compared with healthy controls [10]. Recent prospective metabolomic profiling studies in ARDS have demonstrated that elevated plasma kynurenine levels and increased kynurenine-to-tryptophan ratios are independently associated with higher hospital mortality, suggesting activation of the kynurenine pathway as a potential prognostic marker in ARDS [11]. In the alveolar microenvironment, untargeted mass spectrometry of bronchoalveolar lavage fluid (BALF) from ARDS patients reveals a stark elevation of local lactate, succinate, and purine degradation products, suggesting an association between bioenergetic failure within lung structural cells to the severity of physiological gas exchange degradation [12]. Collectively, these human data show that both excessive metabolic activation and metabolic insufficiency can lead to organ dysfunction through distinct but converging mechanisms.

Mitochondria occupy a central role in this biological framework. As regulators of adenosine triphosphate (ATP) generation, reactive oxygen species (ROS) production, and innate immune signalling, mitochondria integrate metabolic and inflammatory pathways. Mitochondrial dysfunction leads to reduced ATP synthesis, increased oxidative stress, and activation of inflammasome pathways such as the NOD-,LRR- and pyrin domain-containing protein 3 (NLRP3) inflammasome, thereby perpetuating epithelial-endothelial barrier injury even after the initial inflammatory trigger subsides [7, 8]. This establishes a vicious cycle in which bioenergetic failure sustains inflammation and impairs tissue repair.

In addition to intracellular bioenergetics, extracellular metabolites such as lactate, succinate, ATP, and nicotinamide adenine dinucleotide (NAD⁺) derivatives act as signalling mediators that strongly correlate with changes in immune tone, vascular permeability, and reparative responses. These immunometabolites translate cellular metabolic stress into organ-level physiological impairment and this may explain the fluctuating clinical course which is often seen in ARDS [6, 13]. Collectively, these findings suggest a shift from viewing ARDS solely as an inflammatory disorder to recognizing it as a dynamic crisis of metabolic adaptability.

We propose the concept of metabolic resilience, defined as the capacity of the host to restore coordinated cellular energy production, substrate utilization, and redox homeostasis following inflammatory stress, as a unifying framework to explain ARDS heterogeneity. Within this framework, the key determinant of outcome is not simply the magnitude of the inflammatory insult but the ability of immune, epithelial, and endothelial cells to re-establish effective bioenergetic control during recovery. Differences in this adaptive capacity may account for divergent clinical trajectories, variability in therapeutic responsiveness, and the emergence of distinct biological phenotypes in ARDS [5, 6, 9, 14].

### Search strategy and selection criteria

This narrative review was conducted using a structured approach to identify relevant literature on immunometabolic reprogramming, mitochondrial dysfunction, metabolic resilience, and metabolite signalling in ARDS. Relevant literature was identified through searches of major biomedical databases, including PubMed/MEDLINE, with supplementary screening of reference lists from selected articles and key review papers. The search strategy incorporated combinations of keywords such as “acute respiratory distress syndrome”, “ARDS”, “immunometabolism”, “metabolic reprogramming”, “mitochondrial dysfunction”, “metabolic resilience”, “metabolomics”, “bioenergetics”, “oxidative phosphorylation”, “lactate”, “succinate”, and “NAD⁺”. Emphasis was placed on contemporary translational studies, multi-omic investigations, mechanistic experimental studies, clinical cohort analyses, and major review articles relevant to immunometabolic and mitochondrial mechanisms in ARDS. Original research articles, translational investigations, and high-quality narrative and systematic reviews were considered. Studies lacking direct relevance to ARDS immunometabolism or mitochondrial biology were excluded. Given the narrative nature of this review, formal risk-of-bias assessment, systematic evidence quality grading and quantitative synthesis were not performed. Instead, studies were selected based on scientific relevance, methodological rigor, and contribution to the conceptual understanding of metabolic resilience, bioenergetic dysfunction, and phenotype heterogeneity in ARDS.

## Conceptual framework of metabolic resilience

To establish its conceptual novelty, metabolic resilience must be explicitly distinguished from related bioenergetic constructs. Metabolic flexibility traditionally defines a healthy cell’s homeostatic capacity to switch between glucose and lipid oxidation based on substrate availability under physiological conditions [15]. Mitochondrial reserve capacity represents a static cellular parameter, specifically, the measurement of an existing bioenergetic safety margin between baseline ATP production and maximal oxidative capacity under acute demand [8]. In contrast, metabolic resilience operates as a dynamic, systems-level property during overwhelming critical illness stress. It describes not merely a single cellular endpoint, but the host’s multi-layered capacity to actively preserve gas exchange during severe inflammatory stress, survive a temporary bioenergetic down-regulation without progressing to irreversible structural cell apoptosis, and subsequently orchestrate a synchronized metabolic shift back to oxidative phosphorylation to drive energy-intensive barrier repair [16]. Thus, while existing terms define localized, baseline metabolic traits, metabolic resilience captures the broader, time-dependent survival continuum mapping a patient’s trajectory from acute bioenergetic adaptation to systemic failure. The conceptual lineage of metabolic resilience originates from established frameworks of biological systems robustness and the adaptive metabolic downsizing models pioneered by Singer et al., who demonstrated that multi-organ failure can represent an endocrine-mediated, bioenergetic conservation mechanism to survive overwhelming systemic stress [16]. Supportive evidence for metabolic resilience as a potential clinical trajectory exists across both ARDS and non-ARDS conditions. Translational studies in systemic sepsis show that immune cell mitochondrial function is severely inhibited in proportion to disease severity; survivors may be distinguished from non-survivors by their capacity to restore mitochondrial bioenergetic control over time [17, 18]. Similarly, longitudinal monitoring in human ARDS cohorts reveals that patients who successfully clear lactate and restore oxidative profiles over the first week have significantly improved survival rates, whereas individuals displaying persistent metabolic failure face chronic illness and high mortality [18]. To transition this framework into a testable clinical construct, metabolic resilience can be measured at the bedside via dynamic lactate clearance kinetics and longitudinal shifts in the plasma nicotinamide adenine dinucleotide to reduced nicotinamide adenine dinucleotide (NAD^+^/NADH) ratio [19, 20]. At the translational level, it is quantified using patient peripheral blood cells to calculate the Bioenergetic Health Index (BHI), a verified metric measuring mitochondrial reserve capacity under inflammatory stress [21]. This clinical timeline, tracking the divergence between adaptive recovery and progressive bioenergetic failure, is visually synthesized in Fig. 2. Metabolic resilience therefore refers not to the maintenance of baseline function, but the capacity to flexibly reprogram energy production, substrate utilization, and redox balance in response to inflammatory injury and subsequently facilitate a coordinated shift back toward oxidative phosphorylation during recovery [8, 15, 22].

In lung, this resilience requires synchronized regulation of immune activation, mitochondrial energy generation, endothelial barrier integrity, and epithelial repair. Each of these processes is energetically demanding and tightly interdependent. When metabolic coordination is preserved, the lung can tolerate substantial inflammatory stress without progressing to irreversible structural injury. Conversely, failure of adaptive metabolic responses leads to metabolic inflexibility, a state in which cells are unable to appropriately switch between glycolysis and oxidative phosphorylation, resulting in inadequate ATP synthesis, impaired barrier repair, and persistent tissue injury [15].

### Integrated hormonal and nuclear regulation

Metabolic resilience is orchestrated by multilayered hormonal, transcriptional, and epigenetic signalling networks that integrate immune tone with cellular bioenergetic demands. The glucocorticoid receptor alpha (GRα) has been proposed as a central integrator of stress adaptation, influencing mitochondrial biogenesis, substrate utilization, and inflammatory gene transcription [23]. Effective GRα signalling may allow maintenance of gas exchange despite substantial inflammatory stress, whereas impaired receptor responsiveness may predispose to metabolic fragility and organ failure.

At the cellular level, transcriptional and epigenetic regulators further fine-tune metabolic adaptability. Chromatin-remodelling complexes and metabolite-dependent enzymes regulate the expression of genes governing glycolysis, fatty acid oxidation, and mitochondrial respiration. Such regulation highlights that metabolic resilience is not a single-pathway phenomenon but a coordinated systems-level response involving genomic, epigenetic, and endocrine control mechanisms [24, 25].

### Bioenergetic cost of lung injury and repair

All reparative processes in the injured lung are highly energy dependent. Maintenance of the alveolar-capillary barrier requires active sodium transport through sodium-potassium ATPase (Na⁺/K⁺-ATPase) pumps for facilitating alveolar fluid clearance, while surfactant synthesis and membrane repair depend on ATP-intensive lipid biosynthesis pathways [26]. During early inflammatory phases, cells often shift toward rapid glycolysis to meet acute energy and biosynthetic demands. Although initially adaptive, sustained dependence on glycolysis leads to less ATP generation per molecule of glucose and promotes accumulation of lactate and ROS, ultimately compromising energy-intensive reparative processes [27].

Therefore, ARDS can be conceptualized as a condition in which the bioenergetic landscape is temporarily reprogrammed toward short-term survival programs at the expense of long-term functional recovery. Patients who successfully transition back to mitochondrial oxidative metabolism after resolution of the acute insult are more likely to restore alveolar fluid clearance and barrier integrity. In contrast, patients unable to re-establish the oxidative phosphorylation remain trapped in a metabolically inefficient state, predisposing to persistent lung injury, prolonged ventilation, and delayed recovery [28–30]. 

### Metabolic fragility and host susceptibility

Pre-existing host characteristics strongly influence adaptive metabolic capacity. Patients with metabolic syndrome, mitochondrial dysfunction, sarcopenia, or advanced age often exhibit reduced mitochondrial reserve and impaired substrate-switching. In such metabolically fragile patients, even moderate inflammatory insults can trigger disproportionate bioenergetic crises, accelerating progression to ARDS and subsequent multiorgan failure. This variability explains why similar injurious stimuli lead to widely divergent clinical outcomes [17, 23].

Conversely, metabolically robust individuals may tolerate severe inflammatory stress without developing persistent organ failure, owing to preserved mitochondrial function and efficient redox buffering. These observations underscore that ARDS severity is determined not only by the magnitude of the precipitating insult but also by the underlying metabolic terrain of the patient.

Collectively, this conceptual framework positions metabolic resilience as a dynamic systems property integrating immune activation, mitochondrial function, endocrine regulation, and reparative capacity. Failure of this coordinated response results in metabolic inflexibility, bioenergetic failure, and sustained inflammatory injury, thereby providing a mechanistic basis for the biological heterogeneity observed in ARDS [8, 17, 25].

### Sex-based divergence and comorbidity-driven metabolic programming

Pre-existing host characteristics establish distinct baseline biological programs that significantly influence metabolic resilience during critical illness. Sex-based differences in immunometabolism represent an important and often underrecognized contributor to variability in inflammatory and bioenergetic responses. Experimental and translational studies suggest that estrogen signalling enhances mitochondrial biogenesis, stabilizes mitochondrial membrane potential, and limits mitochondrial ROS (mtROS) generation under inflammatory stress [31, 32]. Mechanistically, these effects are mediated in part through estrogen receptor-dependent regulation of peroxisome proliferator-activated receptor gamma coactivator-1 alpha (PGC-1α), a key transcriptional regulator of mitochondrial biogenesis and oxidative metabolism [32]. Such hormonal influences may contribute to greater preservation of mitochondrial function and metabolic adaptability during ALI.

Conversely, critical illness in male patients is frequently associated with profound disturbances in androgen signalling. Clinical studies demonstrate that severe sepsis and acute respiratory failure are commonly accompanied by marked reductions in circulating testosterone levels, which correlate with increased illness severity and adverse clinical outcomes [33]. Emerging evidence further suggests that androgen signalling may modulate innate immune-cell activation, inflammatory cytokine responses, and immunometabolic programming during systemic inflammatory stress [34]. Together, these findings support the concept that sex hormones influence both immune regulation and mitochondrial homeostasis, thereby contributing to inter-individual differences in metabolic resilience during ARDS.

In parallel, chronic comorbid conditions substantially shape the host metabolic terrain before the onset of critical illness. Metabolic syndrome and diabetes promote chronic low-grade inflammation, mitochondrial stress, and impaired substrate-switching capacity, thereby reducing mitochondrial reserve and increasing susceptibility to bioenergetic failure during acute inflammatory insults [7, 17]. Similarly, chronic obstructive pulmonary disease (COPD) and long-term tobacco smoke exposure are associated with persistent oxidative stress and mitochondrial structural abnormalities within airway and alveolar cells [7]. Consequently, patients entering ARDS possess biologically distinct baseline metabolic states that may influence inflammatory trajectories, reparative capacity, and response to metabolism-targeted therapies.

## Immunometabolic reprogramming in lung injury

Activation of innate immune responses during ARDS is closely coupled with profound metabolic reprogramming. When macrophages encounter inflammatory stimuli, they shift from mitochondrial oxidative phosphorylation to aerobic glycolysis, a phenomenon called as the “Warburg effect.” This metabolic transition enables rapid ATP generation and provides biosynthetic intermediates required for cytokine production and effector functions. Thus, cellular metabolism does not merely support immune activation, it is tightly coupled with the magnitude and persistence of the inflammatory response [22, 29].

During this glycolytic shift, the tricarboxylic acid (TCA) cycle becomes functionally disrupted, leading to accumulation of intermediate metabolites such as citrate and succinate. Citrate serves as a precursor for synthesis of inflammatory mediators, whereas succinate stabilizes hypoxia-inducible factor-1α (HIF-1α), promoting transcription of pro-inflammatory cytokines including interleukin-1β (IL-1β) [29]. These metabolites therefore function as signalling mediators that reinforce inflammatory pathways and sustain immune activation even in the absence of ongoing pathogen exposure.

Neutrophils, which are key drivers of early lung injury, also rely predominantly on glycolysis for their energy requirements. This metabolic profile supports rapid chemotaxis, phagocytosis, degranulation, and neutrophil extracellular trap (NET) formation. However, excessive and sustained glycolytic activation enhances ROS production and protease release, leading to epithelial and endothelial injury and worsening gas exchange [35, 36].

Structural lung cells exhibit similar metabolic adaptations, where injured structural lung cells shift to a glycolytic phenotype. This persistent metabolic switch alters local endothelial cytoskeletal dynamics and compromises capillary barrier tightness, accelerating the hyperinflammatory subphenotype transitions discussed below [9, 37]. Endothelial cells likewise undergo metabolic shifts that promote cytoskeletal contraction and increased vascular permeability, which are central features of ARDS progression.

**Fig. 1:**
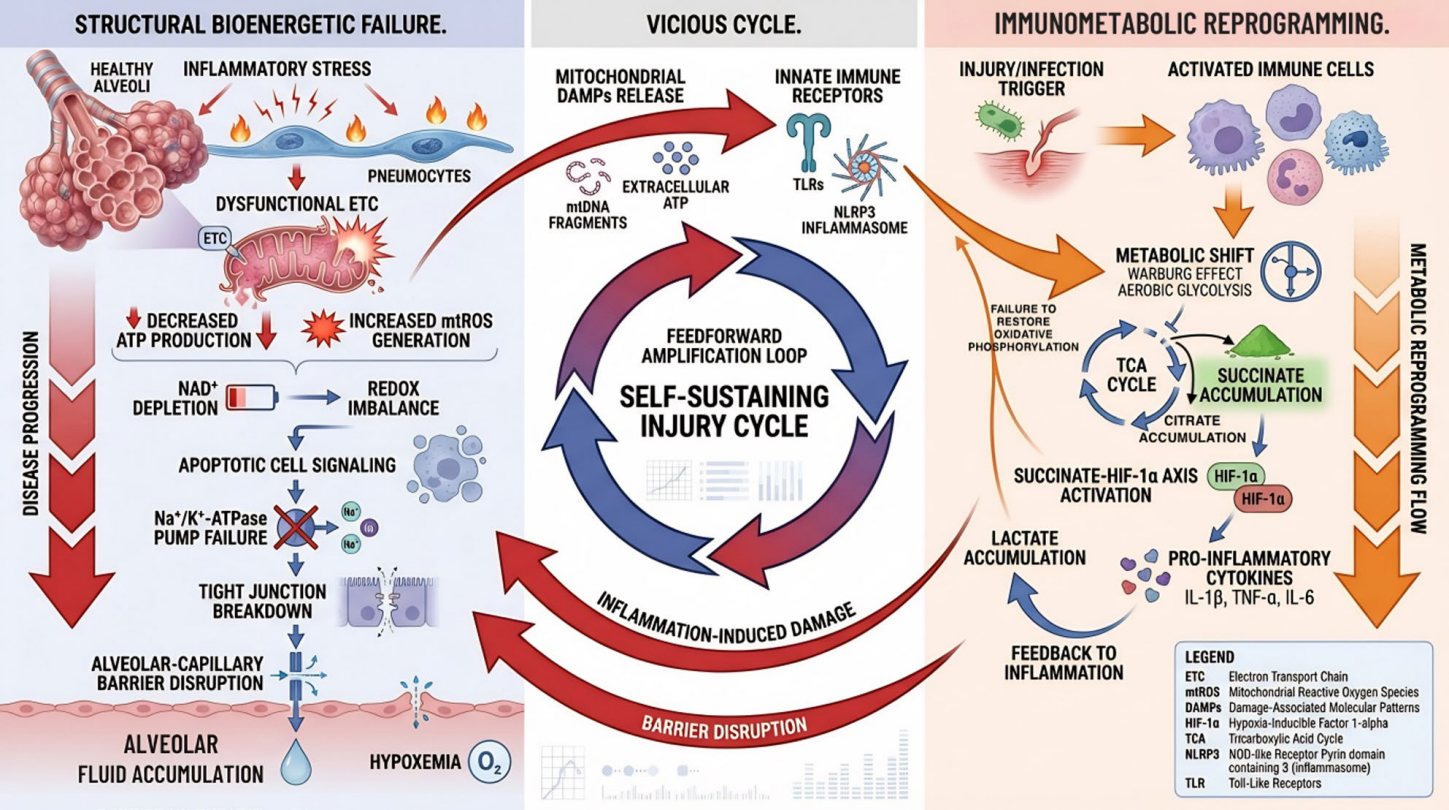
Immunometabolic reprogramming and mitochondrial bioenergetic failure drive a self-sustaining injury cycle in ARDS. Innate immune activation induces glycolytic reprogramming, while inflammatory stress in epithelial and endothelial cells causes mitochondrial dysfunction with ATP depletion, mtROS excess, and NAD⁺ depletion. These processes impair alveolar-capillary barrier integrity and alveolar fluid clearance. Mitochondrial damage releases mtDNA and ATP, that activate innate immune receptors, reinforcing glycolytic inflammation and perpetuating a feedforward cycle of lung injury in ARDS. ARDS: Acute respiratory distress syndrome; ATP: Adenosine triphosphate; ETC: Electron transport chain; mtDNA: Mitochondrial DNA; mtROS: Mitochondrial reactive oxygen species; NAD⁺: Nicotinamide adenine dinucleotide; TLR: Toll-like receptor; TCA: Tricarboxylic acid

These coordinated metabolic changes may help explain the observed differences between ARDS phenotypes. Hyperinflammatory phenotypes are often characterized by sustained glycolytic dominance, increased oxidative stress, and amplified cytokine production. In contrast, hypo inflammatory phenotypes may represent states of metabolic exhaustion with reduced mitochondrial reserve capacity and impaired ability to sustain reparative responses [9, 37]. Although these phenotypes appear distinct, they may reflect a shared underlying disturbance in the regulation of immunometabolic transitions over time. This imbalance likely arises from the interplay between immune metabolic reprogramming and mitochondrial bioenergetic dysfunction, which together sustain inflammatory signalling and contribute to ongoing barrier injury (Fig. 1).

A key determinant of recovery is the ability of immune and structural lung cells to shift from early glycolytic metabolism back to mitochondrial oxidative phosphorylation during the resolution phase of injury. This adaptive capacity, often referred to as ‘metabolic plasticity’, enables suppression of pro-inflammatory signalling and supports tissue repair and restoration of barrier integrity. When oxidative metabolism is not re-established, inflammatory activation persists, ATP production remains inefficient, and progressive bioenergetic dysfunction develops, allowing lung injury to continue despite control of the initial insult [22, 36].

Overall, immunometabolic reprogramming is an early adaptive response to inflammatory stress but becomes harmful when it persists. Short-term metabolic switching may support patient’s defence and cellular adaptation. However, sustained reliance on glycolysis together with impaired mitochondrial oxidative phosphorylation leads to bioenergetic failure and ongoing inflammatory injury. The combined effects of metabolic rewiring and mitochondrial dysfunction across macrophages, neutrophils, alveolar epithelial cells, and pulmonary endothelial cells therefore represent a key mechanism linking inflammatory activation to disruption of the alveolar-capillary barrier and the diverse clinical trajectories observed in ARDS (Table 1).

**Table 1:** Immunometabolic and mitochondrial mechanisms driving lung injury in ARDS

Cellular Compartment	Dominant Metabolic Alteration	Key Metabolic Mediators	Resulting Cellular Dysfunction	Pathophysiological Consequence in ARDS
Macrophages	Shift from oxidative phosphorylation to aerobic glycolysis	Citrate and Succinate accumulation, HIF-1α stabilization	Sustained cytokine production and inflammatory activation	Amplified and persistent lung inflammation
Neutrophils	Predominant reliance on glycolysis	Excess ROS production, NET formation	Oxidative epithelial and endothelial injury	Worsening alveolar damage and impaired gas exchange
Alveolar epithelial cells	Reduced mitochondrial respiration with glycolytic dependence	Decreased ATP availability, mitochondrial dysfunction	Impaired ion transport and fluid clearance	Persistent alveolar flooding and delayed recovery
Pulmonary endothelial cells	Metabolic stress with mtROS generation	RhoA-ROCK signalling activation	Cytoskeletal contraction and increased permeability	Capillary leak and alveolar-capillary barrier disruption

Immunometabolic and mitochondrial alterations across key immune and structural lung cells contributing to alveolar-capillary barrier injury in ARDS. The table summarizes dominant metabolic shifts, principal molecular mediators, and the resulting cellular dysfunction that collectively drive inflammatory amplification, epithelial injury, and endothelial permeability in ARDS

ARDS: Acute respiratory distress syndrome; ATP: Adenosine triphosphate; HIF-1α: Hypoxia-inducible factor-1 alpha; NET: Neutrophil extracellular trap; ROS: Reactive oxygen species; mtROS: Mitochondrial reactive oxygen species; RhoA-ROCK: RhoA-Rho-associated coiled-coil containing protein kinase

## Mitochondrial dysfunction and bioenergetic failure

Mitochondria play a central role in maintaining cellular energy production, redox balance, and regulation of innate immune signalling. In ARDS, sustained inflammatory stress disrupts electron transport chain (ETC) function, leading to reduced ATP generation and increased production of mtROS. While moderate mtROS production contributes to antimicrobial defence and signalling, excessive mtROS causes oxidative damage to mitochondrial deoxyribonucleic acid (mtDNA), lipids, and proteins which further impairs oxidative phosphorylation and amplifies inflammatory signalling pathways [8, 38, 39].

Loss of mitochondrial membrane-potential during severe stress can trigger opening of the mitochondrial permeability transition pore, resulting in release of cytochrome-c and activation of intrinsic apoptotic pathways. Apoptosis of alveolar epithelial and endothelial cells compromises barrier integrity, leading to increased permeability, alveolar flooding, and worsening hypoxemia which are the hallmark features of ARDS progression [40]. These events may contribute to physiological deterioration in gas exchange.

Mitochondrial damage also promotes activation of inflammasome pathways. Release of mtDNA into the cytosol stimulates innate immune receptors and enhances production of IL-1β and interleukin-18 (IL-18), thereby sustaining inflammatory injury even after the initial precipitating insult has been controlled [41]. Circulating mitochondrial damage-associated molecular patterns (mtDAMPs) may further propagate systemic inflammation and contribute to multiorgan dysfunction frequently observed in severe ARDS cases [42, 43].

Bioenergetic failure has immediate physiological consequences in the injured lung. This bioenergetic failure directly compromises the high ATP demands required for active epithelial ion transport and sodium-potassium ATPase pump mechanics [26]. When mitochondrial respiration is suppressed, the lack of immediate ATP causes these active transport networks to fail, culminating in alveolar flooding and severe gas exchange degradation [26, 40]. Similarly, mitochondrial oxidative stress within endothelial cells disrupts cytoskeletal organization and increases vascular permeability through RhoA-Rho-associated coiled-coil containing protein kinase (RhoA-ROCK) signalling pathways, thereby exacerbating capillary leak and lung injury [44].

An important determinant of clinical outcome is mitochondrial reserve capacity, defined as the ability of cells to augment oxidative phosphorylation in response to stress. Patients with preserved mitochondrial reserve may tolerate inflammatory injury and also subsequently recover as metabolic homeostasis is restored. In contrast, those with limited reserve capacity are more susceptible to progressive bioenergetic collapse, persistent inflammation, and organ failure [21]. This concept aligns with clinical observations in which some patients initially stabilize but later deteriorate due to delayed metabolic failure rather than ongoing uncontrolled inflammation.

Thus, mitochondrial dysfunction represents a central nexus linking inflammatory stress to impaired energy production, oxidative injury, and failure of epithelial-endothelial barrier repair. Persistent impairment of mitochondrial bioenergetics can lock cells into an inefficient metabolic state, perpetuating inflammation and preventing effective resolution of lung injury. Crucially, this bioenergetic stability is intrinsically tied to upstream mitochondrial quality control networks, including mitophagy, fission-fusion dynamics, and the secretion of mitochondrial-derived vesicles. During acute inflammatory stress, the PTEN-induced kinase 1 (PINK1)/Parkin pathway acts as a vital homeostatic mechanism by selectively targeting damaged, uncoupled mitochondria for autophagic degradation, thereby limiting the intracellular accumulation of harmful ROS within the alveolar space. This clearance pathway operates alongside tight morphological shifts in mitochondrial fission and fusion dynamics [7]. While inflammatory triggers frequently promote excessive mitochondrial fission facilitating the fragmentation and isolation of severely damaged structural networks, arrested or impaired fusion dynamics prevent the collaborative mixing of functional matrix components, accelerating progressive bioenergetic collapse in lung structural cells. Furthermore, under conditions of severe oxidative stress, cells utilize the extrusion of cargo-packed mitochondrial-derived vesicles as a targeted primary disposal mechanism to remove oxidized proteins and lipids before global organelle apoptosis is triggered [7]. When these combined quality control networks become overwhelmed or dysfunctional in the alveolar compartment, the accumulation of fragmented, un-cleared organelles triggers a rapid breakdown in cellular energetics, directly accelerating the feedforward cycle of lung injury and barrier breakdown. Within the framework of metabolic resilience, the ability to preserve or restore mitochondrial function emerges as a key determinant of whether inflammatory lung injury resolves or progresses to refractory ARDS and multiorgan failure [28, 45, 46].

## Metabolites as active signalling mediators

Metabolic intermediates are increasingly recognized not merely as byproducts of cellular stress but as active signalling molecules that regulate immune responses, vascular permeability, and tissue repair. In ARDS, accumulation and extracellular release of metabolites like lactate, succinate, ATP, NAD⁺ derivatives, and itaconate directly influences inflammatory and reparative pathways, thereby linking intracellular bioenergetic disturbances to organ-level physiological dysfunction [6, 45].

Lactate, traditionally viewed merely as a passive marker of tissue hypoxia and anaerobic metabolism, exerts profound non-hypoxic immunomodulatory effects via specific receptor-mediated and epigenetic pathways [19]. In the ARDS microenvironment, accumulated intracellular and extracellular lactate functions as a signalling molecule that binds to the G-protein coupled receptor GPR81 [19]. This signalling may influence macrophage polarization toward a pro-inflammatory M1 phenotype during early injury phases, driving excessive cytokine release, while simultaneously suppressing the migration and effector functions of specific T-cell subsets [41]. Persistent hyperlactatemia reflects a state of sustained glycolytic dominance and impaired mitochondrial clearance, locking immune cells into an unremitting inflammatory tone [19, 47].

Succinate accumulation represents another critical biological signalling axis triggered by disruption of the TCA cycle during macrophage activation [29]. When mitochondrial metabolism becomes impaired, intracellular succinate accumulates and stabilizes HIF-1α by impairing prolyl hydroxylase activity [29]. Stabilized HIF-1α subsequently translocates to the nucleus, promoting transcription of pro-inflammatory cytokines including IL-1β [29]. This succinate-HIF-1α pathway highlights the role of metabolic intermediates as inflammatory signalling mediators capable of sustaining immune activation independently of ongoing infection [29, 45].

Extracellular ATP functions as a classic damage-associated molecular pattern (DAMP) when released from injured or dying alveolar epithelial and endothelial cells [46, 48]. Once in the extracellular space, ATP binds to purinergic P2X7 receptors on immune cells, triggering potassium efflux and directly activating the NLRP3 inflammasome pathway [8, 46]. This activation drives the maturation and secretion of IL-1β and IL-18, severely disrupting alveolar-capillary barrier tight junctions and causing capillary leak [8, 44]. Conversely, the enzymatic conversion of extracellular ATP to adenosine by ectonucleotidases exerts local anti-inflammatory and barrier-protective effects, highlighting the context-dependent signalling role of purinergic networks in lung tissue injury [48, 46].

Itaconate, an endogenous metabolite derived from cis-aconitate within the rewired TCA cycle, serves as a potent counter-regulatory signalling mediator [6]. Synthesized predominantly by activated macrophages, itaconate functions as an intrinsic metabolic brake to prevent runaway tissue destruction [6, 49]. Mechanistically, itaconate directly inhibits succinate dehydrogenase, stopping excessive mtROS generation [49]. Furthermore, it alkylates the protein KEAP1, allowing the master antioxidant transcription factor Nrf2 to translocate to the nucleus and initiate a broad anti-inflammatory and antioxidant cytoprotective gene program [49]. Dysregulation or insufficiency of this protective itaconate signalling pathway contributes directly to unresolving inflammation and delayed recovery in severe ARDS cases [6, 49].

Collectively, these metabolites form an interconnected signalling network that translates cellular metabolic stress into coordinated inflammatory, vascular, and reparative responses. Temporal fluctuations in these immunometabolites may explain the dynamic bedside physiology observed in ARDS cases, where patients may transiently improve and subsequently deteriorate as maladaptive metabolic signalling persists. Within the metabolic resilience framework, effective resolution of lung injury likely depends on restoration of balanced metabolite signalling alongside recovery of mitochondrial bioenergetic control [45–47, 50]. The major metabolite-driven signalling pathways and their roles in ARDS pathobiology are summarized in Table 2.

**Table 2:** Metabolic signalling mediators and their functional roles in ARDS pathophysiology

Metabolite / Signalling Axis	Source of Alteration in ARDS	Principal Biological Effects	Contribution to Disease Progression
Lactate	Sustained glycolytic dominance and impaired mitochondrial respiration	Modulates macrophage polarization, cytokine production, and redox balance	Reflects metabolic stress and correlates with worse outcomes
Succinate-HIF-1α pathway	Disrupted TCA cycle during inflammatory activation	Stabilizes HIF-1α and promotes pro-inflammatory cytokine production	Sustains inflammatory signalling independent of ongoing infection/insult
Extracellular ATP-purinergic signalling	Release from injured or dying cells	Inflammasome activation and increased endothelial permeability	Amplifies lung injury and vascular leak
NAD⁺ homeostasis	Excess consumption during severe inflammatory stress	Regulates mitochondrial respiration, redox balance, and cell survival pathways	Impaired NAD⁺ balance contributes to bioenergetic failure
Itaconate pathway	Endogenous production during macrophage activation	Inhibits succinate dehydrogenase and activates Nrf2 antioxidant responses	Functions as intrinsic brake limiting excessive inflammatory injury

Major metabolite-derived signalling pathways implicated in ARDS pathophysiology. The table outlines key metabolic intermediates, their sources during immunometabolic reprogramming, principal biological effects, and their roles in sustaining inflammation, vascular leak, and bioenergetic imbalance

ARDS: Acute respiratory distress syndrome; ATP: Adenosine triphosphate; HIF-1α: Hypoxia-inducible factor-1 alpha; NAD⁺: Nicotinamide adenine dinucleotide; Nrf2: Nuclear factor erythroid 2-related factor 2; TCA: Tricarboxylic acid

## Multi-omic evidence linking metabolism to ARDS phenotypes

Recent advances in multi-omic technologies have provided compelling evidence that metabolic dysregulation is closely linked to ARDS phenotypes, disease severity, and clinical outcomes. Integrative analyses combining metabolomics, transcriptomic and proteomic data show that distinct metabolic signatures characterize biologically defined ARDS subphenotypes and may contribute to their differential clinical trajectories [10, 51, 52].

### Plasma metabolomics

Plasma metabolomic profiling in critically ill patients demonstrated that non-survival was associated with enhanced glycolytic intermediates, impaired fatty acid β-oxidation, and increased amino acid catabolism, suggesting profound bioenergetic dysfunction in severe disease states [51]. These metabolic perturbations correlate with higher inflammatory biomarker levels, severe hypoxemia, and increased mortality risk. In contrast, hypoinflammatory phenotypes often display metabolic profiles suggestive of relative metabolic quiescence or exhaustion, with lower levels of glycolytic intermediates but also reduced oxidative metabolic capacity [9, 53].

Metabolomic analyses in sepsis-induced ALI revealed disruption of pathways associated with oxidative stress, apoptosis, endothelial barrier integrity, and energy metabolism. Specifically, altered levels of total glutathione, adenosine, phosphatidylserine, and sphingomyelin reflected key biological processes implicated in lung injury pathogenesis [10]. These findings provide biochemical evidence linking systemic metabolic derangements to endothelial injury and impaired tissue oxygen utilization in ARDS.

### BALF metabolomics

Metabolomic profiling of BALF offers direct insight into lung-specific metabolic alterations. Studies show that BALF from ARDS patients contains elevated levels of lactate, succinate, and purine metabolites, indicating local glycolytic activation, TCA-cycle disruption, and ATP degradation within the alveolar microenvironment [12]. These lung-specific metabolic signatures can differentiate ARDS from other causes of respiratory failure and correlate with severity of lung injury and clinical outcomes.

Importantly, BALF and plasma metabolomes exhibit only partial overlap, suggesting that local pulmonary and systemic metabolic perturbations represent distinct but complementary aspects of disease biology. This compartmentalized metabolic heterogeneity implies that comprehensive phenotyping of ARDS may require simultaneous assessment of both systemic and lung-specific metabolic states [54]. However, translating this spatial complexity into routine critical care practice remains challenging, particularly because serial BALF sampling is often clinically impractical due to the risks of alveolar derecruitment, barotrauma, and worsening hypoxemia. To address this limitation, a pragmatic two-tier compartmental sampling framework may be considered. During the early acute phase of illness, particularly within the first 24 h of mechanical ventilation, clinicians may prioritize localized pulmonary sampling using non-directed bronchial lavage or minimally invasive endotracheal aspirates to characterize compartment-specific metabolic biology and help distinguish native ARDS bioenergetic profiles from alternative causes of acute respiratory failure. In contrast, for longitudinal bedside monitoring, peripheral plasma sampling may serve as the preferred compartment because of its minimally invasive and repeatable nature. Serial plasma metabolomic profiling could therefore be used to track dynamic biomarkers, including lactate clearance kinetics and temporal alterations in circulating acylcarnitines or NAD⁺ pools, which may function as accessible surrogate indicators of evolving metabolic resilience or progressive multiorgan bioenergetic failure.

### Longitudinal metabolic trajectories

Longitudinal metabolomic studies indicate that dynamic metabolic trajectories during the early phase of ARDS are more informative than single time-point measurements. Patients who show normalization of lactate levels and restoration of oxidative metabolic profiles over the first week of critical illness, tend to have improved survival. In contrast, those with persistent metabolic dysregulation exhibit sustained inflammatory activation, ongoing mitochondrial dysfunction, and higher mortality. However, it must be noted that robust trajectory data derive predominantly from systemic sepsis populations rather than native human ARDS cohorts. In unselected sepsis and general critical care cohorts, the prognostic value of dynamic metabolic tracking is firmly quantified. For instance, Langley et al. analysed multicentre derivation and validation cohorts comprising 1,152 septic patients with acute respiratory failure and identified persistent dysregulation of glycolytic intermediates, acylcarnitines, and amino acid metabolism as strong correlates of adverse mortality trajectories [51]. Similarly, longitudinal lactate clearance studies demonstrated that failure to reduce lactate levels during early critical illness was independently associated with increased mortality risk [55]. In contrast, direct ARDS-specific longitudinal metabolomic studies remain comparatively small and observational. Evans et al. evaluated BALF metabolomic profiles in ARDS patients and demonstrated persistent accumulation of lactate, succinate, and purine degradation products in association with severe and prolonged lung injury [12]. Collectively, these findings suggest that dynamic metabolic profiling may better reflect evolving host bioenergetic states than isolated static measurements. Nevertheless, current evidence remains predominantly associative, heterogeneous across platforms, and partially extrapolated from sepsis biology. Large prospective ARDS-specific longitudinal studies with serial multi-omic sampling are therefore required before dynamic metabolic trajectories can be validated for routine prognostication or precision-guided therapeutic stratification.

### Integration with clinical phenotypes

Multi-omic integration studies combining metabolomics, transcriptomics, and proteomics have identified distinct metabolic endotypes nested within established clinical phenotypes. For example, within the hyperinflammatory ARDS phenotype, subgroups with divergent metabolic profiles demonstrate differential responses to fluid management strategies and pharmacologic interventions [37, 56]. Such findings highlight that clinical phenotypes alone do not fully capture underlying biological heterogeneity and that metabolism-informed endotyping may improve therapeutic targeting.

The unique predictive and therapeutic value of metabolic endotyping lies in its capacity to identify hidden, intracellular bioenergetic states nested within identical clinical subphenotypes. While latent class analysis effectively splits patients into broad hyper- and hypo-inflammatory classes, these groups remain biologically heterogeneous. For instance, two patients matching the exact same hyperinflammatory clinical subphenotype can possess opposite metabolic configurations: one exhibiting a highly active, glycolysis-driven immune response requiring an immunometabolic brake, while another has progressed to secondary mitochondrial collapse requiring bioenergetic support. As established by secondary modelling of major clinical trials, treating these unselected biological trajectories under a single phenotype can hide true therapeutic signals [56, 57]. Therefore, metabolic endotyping provides distinct clinical utility beyond standard subphenotyping by targeting the precise enzymatic and substrate handling defects that dictate whether a patient will recover or progress to refractory organ failure.

Current multi-omic investigations in critical illness remain predominantly correlative rather than causal in nature. In a large multicentre metabolomic study involving 1,152 patients with community-acquired sepsis, Langley et al. used liquid chromatography-mass spectrometry (LC-MS) to identify metabolic alterations associated with mortality trajectories and subsequently derived a prognostic biomarker model integrating clinical variables and metabolite signatures [51] utilized a large multi-centre study population of septic patients (*n* = 1,152 total across derivation and validation cohorts) presenting with severe sepsis and associated acute respiratory failure. Using LC-MS, they successfully identified a 44-metabolite panel that correlated strongly with mortality trajectories. Similarly, in lung-specific sampling, Evans et al. [12] evaluated BALF from a smaller, single-centre population of established ARDS cases (*n* = 18) against healthy controls (*n* = 8), showing localized accumulation of glycolysis and purine degradation products via untargeted LC-MS. While these high-throughput profiling studies are methodologically robust for biomarker discovery and patient stratification, they remain inherently descriptive and associative. Mechanistic causality, proving that specific metabolic pathways directly drive alveolar barrier injury or resolution, stems primarily from preclinical animal models and in vitro genetic interventions, such as macrophage-specific knockouts demonstrating that succinate accumulation directly induces IL-1β transcription via normoxic HIF-1α stabilization. Significant evidence gaps remain before these multi-omic findings can be translated into routine bedside practice. First, almost all existing human metabolomic data rely on bulk plasma or bulk BALF sampling. This approach averages out the biological signals and obscures critical cell-type-specific metabolic heterogeneity inside the injured alveolus, effectively masking the distinct bioenergetic profiles of alveolar type II cells versus infiltrating neutrophils. Second, current clinical multi-omic studies are predominantly cross-sectional or limited to a single time point during early intensive care unit (ICU) admission, which may not fully capture the highly dynamic shifts that characterize the timeline of clinical lung injury. To bridge these gaps, future research must shift toward integrating single-cell transcriptomics and spatial metabolomics in human samples to map metabolic pathways within specific cell compartments. Such approaches may substantially improve the precision of biomarker discovery and reveal compartment-specific metabolic endotypes that are obscured by conventional bulk analyses. Additionally, clinical trial designs should incorporate serial, longitudinal multi-omic sampling across multi-centre cohorts to clearly validate the predictive power of dynamic metabolic trajectories over static baseline measurements.

**Fig. 2:**
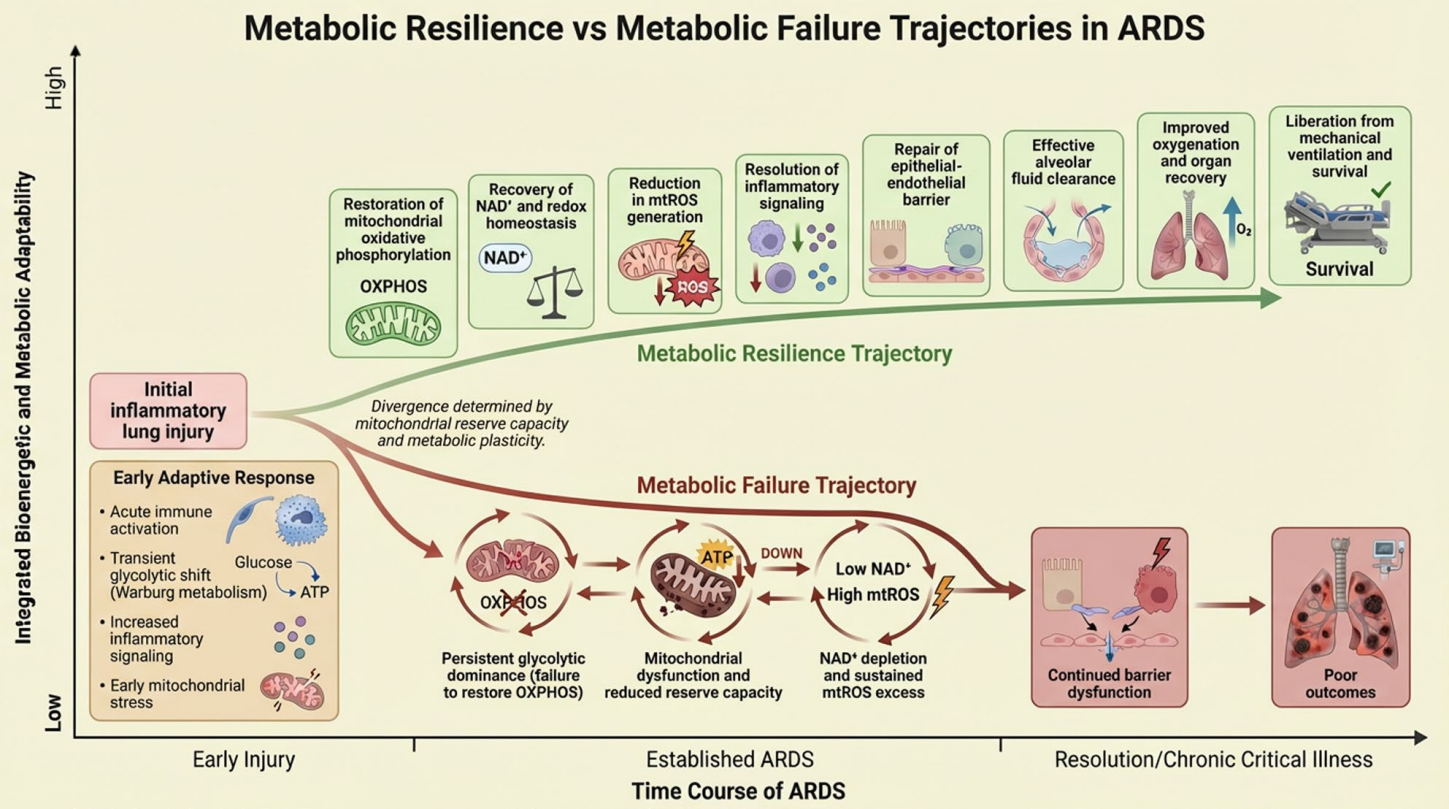
Metabolic resilience versus metabolic failure trajectories in ARDS. Following the initial inflammatory lung injury and transient glycolytic activation, patients diverge into two possible trajectories. Metabolic resilience involves restoration of mitochondrial oxidative phosphorylation, redox homeostasis, barrier repair, and clinical recovery. Metabolic failure is marked by persistent glycolytic dominance, mitochondrial dysfunction, NAD⁺ depletion, and sustained barrier injury, leading to refractory hypoxemia, chronic critical illness and mortality. ARDS: Acute respiratory distress syndrome; mtROS: Mitochondrial reactive oxygen species; NAD⁺: Nicotinamide adenine dinucleotide; OXPHOS: Oxidative phosphorylation; CCI: Chronic critical illness

Overall, multi-omic evidence strongly supports the concept that metabolic dysregulation is not merely an epiphenomenon but a central biological axis driving phenotype divergence, disease progression, and treatment responsiveness in ARDS. By linking systemic and lung-specific metabolic changes with longitudinal clinical trajectories, these studies support metabolic resilience as a unifying framework for understanding heterogeneity in ARDS outcomes, where patients may progress toward either adaptive bioenergetic recovery or persistent metabolic failure over time as presented in Fig. 2 [52–54, 58]. The primary metabolic biomarkers, phenotype-associated metabolomic signatures, concrete clinical cohort characteristics, and associated prognostic metrics are detailed comprehensively in Table 3.

**Table 3:** Clinical studies evaluating metabolic biomarkers and metabolomic signatures in ARDS and sepsis-associated acute lung injury

Biomarker class	Cohort / study design	*n*	Results
Plasma kynurenine / kynurenine-to-tryptophan ratio	Prospective longitudinal ARDS cohort with serial plasma and urine sampling at day 1, 3, and 7; healthy controls included	69 ARDS; 30 controls	Day-1 plasma kynurenine and kynurenine/tryptophan ratio were independently associated with hospital mortality. Non-survivors had higher kynurenine and K/T ratio on all sampling days; a day-1 kynurenine cutoff of 15.12 µM stratified mortality (66.7% vs. 25%). [11]
BALF metabolomics	Single-centre BALF LC-MS study in mechanically ventilated ARDS patients sampled 0–72 h after ARDS diagnosis, compared with healthy controls	18 ARDS; 8 controls	BALF showed higher lactate, succinate, hypoxanthine, xanthine, guanosine, hippurate, O-acetylcarnitine, cis-aconitate, and related metabolites. The study reports differentiating metabolites and pathway enrichment, not a formal AUROC. [12]
Plasma amino acids / biogenic amines in sepsis-induced ALI	Prospective ^1^H-NMR metabolomics pilot study in sepsis-induced ALI versus healthy volunteers	13 ALI; 6 controls	Altered total glutathione, adenosine, phosphatidylserine, sphingomyelin, and myoinositol. Myoinositol was inversely associated with ventilator-free days, and total glutathione correlated positively with APACHE severity score. [10]
Acylcarnitine-containing composite metabolomic panel	Multicentre community-acquired sepsis cohort used as a translational comparator for metabolomic prognostication	1,152 total	A 7-variable model containing four acylcarnitines plus lactate, age, and hematocrit achieved AUC 0.847 and accuracy 85.1% in the discovery cohort, and outperformed SOFA/APACHE II/capillary lactate. [51]

This table summarizes key clinical cohort studies evaluating metabolic biomarkers and metabolomic profiling approaches in ARDS and sepsis-associated ALI. Included studies describe cohort populations, study designs, sample sizes, analytical platforms, and principal diagnostic or prognostic findings related to metabolic dysregulation, inflammatory activation, mitochondrial dysfunction, and clinical outcomes. While several biomarkers demonstrated associations with mortality risk and disease severity, most available evidence remains exploratory and observational, highlighting the need for larger prospective validation studies before routine clinical implementation

ALI: Acute lung injury; APACHE: Acute Physiology and Chronic Health Evaluation; ARDS: Acute respiratory distress syndrome; AUROC: Area under receiver operating characteristic curve; BALF: Bronchoalveolar lavage fluid; LC-MS: Liquid chromatography-mass spectrometry; K/T ratio: Kynurenine-to-tryptophan ratio; NMR: Nuclear magnetic resonance; SOFA: Sequential organ failure assessment

## Metabolic biomarkers and clinical phenotyping

Translation of metabolic insights into clinical practice requires identification of robust, reproducible biomarkers that can be used for real-time phenotyping and prognostication in ARDS. Several metabolic markers have emerged as promising candidates to reflect underlying bioenergetic status, mitochondrial function, and immunometabolic balance, thereby serving as potential clinical surrogates of metabolic resilience [59]. To provide a structured and methodologically rigorous framework for these candidates, the concrete clinical cohort characteristics, study designs, sample sizes, and quantitative diagnostic performance metrics for each major biomarker class are synthesized and presented in Table 3.

### Lactate

Lactate remains the most widely used metabolic biomarker in critical care. The diagnostic and prognostic utility of lactate kinetics has been validated in large, prospective multi-centre clinical cohorts [19, 55]. Elevated lactate levels were associated with sepsis non-survival and were incorporated into metabolite-based prognostic models predicting 28-day mortality [51]. Dynamic assessment of lactate clearance may provide greater prognostic insight than isolated static lactate measurements [55].

### Amino acid profiles

Quantitative plasma metabolomic profiling demonstrated associations between selected metabolites and clinical severity indices in sepsis-induced ALI. Myoinositol levels were inversely associated with ventilator-free days, while total glutathione levels correlated positively with acute physiology scores, suggesting potential links between metabolic disturbances and disease severity [10]. Mechanistically, activation of the kynurenine pathway through indoleamine 2,3-dioxygenase activity contributes to tryptophan degradation, kynurenine accumulation, immune tolerance, and oxidative stress, thereby linking immunometabolic dysregulation with ARDS pathogenesis and progression [11].

### Acylcarnitines

Acylcarnitines are intermediates of fatty acid oxidation and provide insight into mitochondrial substrate handling. The clinical significance of these lipophilic intermediates was rigorously demonstrated via ^1^H-NMR spectroscopy in an observational cohort of critically ill patients [10, 60]. This pilot metabolomic study identified distinct alterations in plasma metabolites among patients with sepsis-induced ALI compared with healthy controls, supporting the concept that metabolic profiling may aid in identifying biomarkers associated with disease severity and pathophysiological processes in ALI [10].

### NAD⁺ and related metabolites

NAD⁺ and its related metabolites, including nicotinamide and nicotinamide mononucleotide, are essential for mitochondrial respiration, maintenance of redox balance, and regulation of metabolic sensors such as sirtuins [20]. Reduced NAD⁺ levels and altered NAD⁺/NADH ratios have been dynamically tracked in prospective observational cohorts of critically ill patients (*n* = 45) [20, 61]. Quantitative platform measurements show that severe depletion of the systemic NAD⁺ pool within the first 72-hours of critical illness correlates significantly with progressive multi-organ dysfunction syndrome severity scores and a failure to transition cellular metabolism back to oxidative phosphorylation (*p* < 0.05). These clinical cohort findings reveal that differences in the host’s ability to preserve or actively restore NAD⁺ homeostasis serve as a direct metabolic reflection of underlying metabolic resilience and susceptibility to persistent alveolar barrier injury [20, 61].

### Composite metabolomic signatures

Machine learning approaches applied to comprehensive metabolomic datasets have identified multi-metabolite signatures that outperform single biomarkers for ARDS phenotype classification and mortality prediction [18, 51]. In multi-centre validation cohort designs analysing large patient populations (*n* = 1,152), composite signatures integrating markers of glycolysis, TCA-cycle function, amino acid metabolism, fatty acid oxidation, and oxidative stress achieved superior prognostic accuracy for predicting 28-day survival trajectories compared to classic clinical severity scores alone, demonstrating a high diagnostic performance with an area under receiver operating characteristic curve of 0.84–0.88 [18, 51, 57]. The integration of these high-dimensional metabolomic signatures with clinical variables enables precise, biology-driven patient stratification for clinical trials and targeted metabolic therapies [57].

### Challenges in clinical implementation

Despite promising findings, several core methodological challenges limit the routine translation of metabolic biomarkers to the bedside. First, current metabolomic platforms differ considerably in metabolite coverage, analytical performance, and reproducibility, leading to significant cross-study variability [62]. Second, sample collection parameters introduce substantial pre-analytical confounding; factors such as the timing of sample collection, precise extraction processing methods, and ultra-low storage conditions drastically affect intermediate stability and measured concentrations [62, 63]. Furthermore, the majority of current metabolomic validation studies are restricted to small (*n* < 100), single-centre cohorts lacking external geographic validation, which raises significant concerns regarding the generalizability of these metabolic classifiers [57, 62]. Consequently, before these high-dimensional signatures can be formally proposed as clinical biomarkers, future multi-centre consortiums must subject these datasets to rigorous, standardized quality appraisal frameworks, such as the Grading of Recommendations Assessment, Development and Evaluation (GRADE) criteria, to objectively quantify the certainty of evidence. Finally, mass spectrometry and nuclear magnetic resonance platforms require highly specialized laboratory equipment and prolonged processing turnaround times, making them structurally unsuitable for real-time bedside decision-making. Overcoming these barriers requires the development of standardized point-of-care assays for key target intermediates, validation of parsimonious biomarker panels across large multi-centre populations, and integration with machine learning-based classifiers before metabolic phenotyping can be safely translated into routine critical care practice [57, 62–64].

Overall, metabolic biomarkers offer a promising approach to bridge mechanistic insights with bedside phenotyping. By showcasing underlying mitochondrial function, substrate utilization, and immunometabolic balance, these markers may enable early identification of patients with impaired metabolic resilience who are at higher risk of persistent lung injury and poor outcomes in ARDS [11, 57, 59–62].

## Therapeutic implications and emerging interventions

If dysregulated metabolic signalling plays a fundamental role in ARDS pathogenesis, therapeutic strategies may need to extend beyond suppression of inflammation and focus on restoring coordinated cellular bioenergetics. Interventions that preserve mitochondrial function, recalibrate immunometabolic pathways, and correct maladaptive metabolite signalling may help interrupt the cycle of inflammation, barrier injury, and organ dysfunction [8, 63].

### Preserving mitochondrial function

Preserving mitochondrial integrity is a compelling therapeutic goal in ARDS, as maintaining ETC function can curb harmful ROS, limit cell apoptosis, and help restore the alveolar barrier. At the preclinical level, mitochondria-targeted antioxidants like mitoquinone (MitoQ) and the cardiolipin-stabilizing peptide SS-31 (elamipretide) have shown excellent results in animal models of ALI [7, 66]. These agents successfully lower mitochondrial oxidative stress and preserve lung tissue architecture. However, their current clinical translational value remains modest because no large-scale human randomized controlled trials (RCTs) have validated them in critically ill patients. Translation is currently bottlenecked by challenges in delivering these drugs directly to the lung tissue, determining the optimal timing during the injury phase, and the lack of reliable bedside biomarkers to tell us which patients are actively experiencing mitochondrial failure [66].

### NAD^+^ repletion strategies

Maintaining adequate cellular NAD^+^ pools is essential for mitochondrial respiration, oxidative stress buffering, and activation of sirtuin-mediated repair pathways. Current evidence supporting NAD^+^ repletion using precursors such as nicotinamide riboside (NR) or nicotinamide mononucleotide (NMN) remains predominantly preclinical [20, 67]. In experimentally induced mouse models of sepsis and endotoxin-mediated lung injury, administration of NR increased intracellular NAD^+^ availability, reduced oxidative stress, attenuated pulmonary microvascular permeability, and decreased inflammatory tissue injury through modulation of NAD^+^/SIRT1 signalling pathways. These protective effects were associated with reduced HMGB1 release, decreased endothelial apoptosis, and improved survival outcomes in septic mice [68]. Despite these promising mechanistic findings, the translational value of NAD^+^ repletion in human ARDS remains uncertain, and future progress will require early-phase clinical trials to determine whether systemic NAD^+^ restoration can safely improve bioenergetic recovery during critical illness.

### Modulating immunometabolism

Modulating maladaptive immunometabolic reprogramming is a direct way to speed up the resolution phase of ARDS by preventing immune cells from getting locked into a highly inflammatory glycolytic state. The clinical evidence here spans a wide mix of observational and experimental data. For the biguanide metformin, the evidence is mostly observational. Large retrospective cohort studies have suggested that pre-admission metformin use may be associated with lower mortality among critically ill diabetic patients, including those with sepsis and septic shock, although ARDS-specific outcome benefits remain uncertain [69]. However, prospective RCTs evaluating metformin in unselected critically ill populations have failed to show a consistent survival benefit, highlighting the classic gap between observational associations and true clinical causality [70]. For newer immunometabolic modulators, like endogenous itaconate derivatives (e.g., 4-octyl itaconate), the data remains entirely preclinical [6, 71]. While these derivatives successfully act as a metabolic brake to calm macrophage inflammation in petri dishes, their translational value is limited by short plasma half-lives and a total lack of human safety data in critical illness [49, 71].

### Nutritional support and metabolic substrates

Optimizing nutritional support and supplying specific metabolic substrates represents a pragmatic, bedside strategy to fuel oxidative pathways and prevent severe muscle and tissue wasting during critical illness. The level of clinical evidence for general nutritional strategies in ARDS spans multiple large, high-quality human RCTs [72, 73]. Landmark trials (such as the EDEN trial) have clearly demonstrated that initial trophic enteral feeding provides similar clinical outcomes, including ventilator-free days and 60-day mortality, compared to full early enteral feeding, while significantly reducing gastrointestinal intolerance [72]. Furthermore, large RCTs evaluating the routine supplementation of specific immunonutritional substrates, such as omega-3 fatty acids, antioxidants, and selenium (e.g., the OMEGA trial), failed to show a clinical benefit and, in some subsets, suggested potential harm [74]. Consequently, while nutritional support has high translational value for general intensive care management, unselected metabolic substrate supplementation carries low therapeutic value for targeted ARDS resolution. Current guidelines recommend avoiding routine immunonutrition, focusing instead on early, well-tolerated trophic enteral feeding [72, 73].

### Corticosteroids

Corticosteroids are systemic anti-inflammatory agents that exert profound, wide-ranging effects on cellular metabolism and immunometabolic signalling. The level of clinical evidence for corticosteroids in ARDS is backed by a combination of clinical trials and international practice guidelines [74, 75]. Preclinical and translational data show that glucocorticoids help suppress the hyper-glycolytic shift in activated immune cells and protect the endothelial barrier from inflammatory degradation. Clinically, while the landmark DEXA-ARDS trial by Villar et al. demonstrated that early dexamethasone in moderate-to-severe ARDS significantly increases ventilator-free days and reduces mortality, it is critical to note that this was a single-centre study, which warrants cautious interpretation amid ongoing clinical debate [74]. Reflecting this biological and trial heterogeneity, the 2024 focused update guidelines offer nuanced, conditional recommendations, balancing potential survival benefits against the metabolic double-edged sword of corticosteroid therapy, such as systemic hyperglycaemia and insulin resistance [75]. Therefore, corticosteroids carry high clinical translational value and represent one of the few established, standard-of-care metabolic interventions in routine clinical practice. However, their metabolic impact remains a double-edged sword, as systemic steroid use frequently induces severe hyperglycaemia and insulin resistance, which can impair systemic metabolic flexibility and worsen muscle weakness if not tightly managed [75].

### Mesenchymal stromal cell (MSC) therapy

MSCs exert broad therapeutic effects, including clearing alveolar fluid, modulating inflammation, and actively transferring functional mitochondria to injured lung cells through tunnelling nanotubes. Crucially, the evidence for MSC therapy in ARDS has successfully moved into human Phase 1 and Phase 2 a randomized clinical trials [76]. Preclinical work has consistently suggested that this mitochondrial transfer may help restore the bioenergetics of injured epithelial cells, thereby improving ATP availability required for alveolar fluid clearance [26, 77]. Recent evidence further suggests that the therapeutic efficacy of MSC-mediated mitochondrial transfer may vary across ARDS phenotypes and underlying metabolic states. A 2024 systematic review and meta-analysis of 17 clinical trials involving 796 patients demonstrated that MSC therapy was associated with reduced all-cause mortality, improved oxygenation indices (PaO₂/FiO₂ ratio), and attenuation of systemic inflammatory markers including interleukin-6 and C-reactive protein [78]. Emerging translational evidence also indicates that the bioenergetic benefits of exogenous mitochondrial transfer may be more pronounced in hypo inflammatory phenotypes characterized by mitochondrial exhaustion and impaired oxidative metabolism, whereas severely hyperinflammatory states dominated by persistent glycolytic activation may exhibit attenuated responsiveness [79]. In addition to mitochondrial donation, MSCs exert broader immunometabolic effects by modulating inflammatory signalling pathways such as nuclear factor -κB(NF-κB) and toll-like receptor 4 (TLR4) while simultaneously activating cytoprotective antioxidant responses including Nrf2-mediated pathways. These observations further support the concept that metabolic and inflammatory heterogeneity may significantly influence therapeutic responsiveness in ARDS and highlight the importance of phenotype-informed stratification in future MSC-based clinical trials.

Clinically, multi-centre safety trials (such as the START trial) have shown that intravenous allogeneic MSCs are highly tolerated, with early signs pointing lower systemic inflammation [76]. Consequently, MSCs carry high clinical translational value. However, definitive clinical efficacy has not yet been proven, and large-scale, Phase 3 predictive enrichment trials are still needed to confirm a true survival benefit across different biological phenotypes [76, 80].

### Precision medicine and metabolic endotype-targeted therapy

Given the substantial metabolic heterogeneity observed in ARDS, a precision medicine approach that matches interventions to specific metabolic endotypes may be necessary to achieve consistent therapeutic benefit. Patients with hyperinflammatory, glycolysis-dominant phenotypes may benefit from therapies that promote metabolic reprogramming toward oxidative metabolism, whereas those with hypo inflammatory, metabolically exhausted phenotypes may require bioenergetic support and mitochondrial rescue strategies [56].

Incorporating metabolic phenotyping into clinical trial design may help identify patient subgroups that are more likely to benefit from specific metabolic interventions. Such metabolism-informed therapeutic stratification aligns with emerging precision critical care approaches and may improve the success of future trials addressing ARDS heterogeneity. Currently, the clinical level of evidence supporting these endotype-targeted strategies rests primarily on retrospective secondary analyses of large clinical trials and predictive enrichment simulations [18, 57]. Secondary modelling consistently demonstrates that ‘one-size-fits-all’ metabolic approaches can cause harm by inadvertently treating opposite biological trajectories such as applying an immunometabolic brake to a patient who is already suffering from severe bioenergetic exhaustion [18, 57]. Importantly, metabolism-targeted therapies must be interpreted within the broader context of repeated translational failures in ARDS and critical illness, including tumor necrosis factor inhibition, IL-1β blockade, statins, antioxidant supplementation, and several metabolic interventions that failed to demonstrate consistent survival benefit in unselected ICU populations [70, 73]. The proposed rationale for metabolic targeting is not that these therapies are inherently superior, but that immunometabolic dysfunction may represent a more upstream, integrated regulator of inflammatory signalling, mitochondrial bioenergetics, redox balance, and tissue repair. Nevertheless, these approaches will likely fail if applied indiscriminately without accounting for the marked biological heterogeneity of ARDS. Consequently, metabolism-targeted therapies should currently be viewed as hypothesis-generating precision strategies requiring rigorous prospective validation and metabolic endotype-guided patient selection rather than established therapeutic interventions. Given the profound biological heterogeneity of ARDS, successful implementation of metabolism-targeted therapies will likely require precision-guided trial designs incorporating metabolic stratification, optimal temporal intervention windows, and adaptive enrichment methodologies. A proposed conceptual framework summarizing candidate metabolism-targeted therapeutic strategies alongside relevant metabolic profiles, suggested therapeutic timing windows, candidate translational endpoints, and potential biologic stratification approaches is presented in Table 4.

**Table 4:** Proposed translational framework for metabolism-informed precision trials in ARDS

Therapeutic Strategy	Proposed Target Population / Metabolic Profile	Suggested Timing Window	Candidate Translational Endpoints	Potential Stratification Approach
Mitochondrial-targeted antioxidants (MitoQ, SS-31)	Patients with evidence of mitochondrial dysfunction and oxidative stress	Early acute/exudative phase	Lactate clearance, oxidative stress markers, organ failure scores	Elevated acylcarnitines, mtROS-associated profiles
NAD⁺ precursor supplementation	Patients with impaired oxidative metabolism and NAD⁺ depletion	Early-to-intermediate phase of critical illness	NAD⁺/NADH ratio, bioenergetic recovery markers	Reduced systemic NAD⁺ metabolomic signatures
MSC-mediated mitochondrial transfer	Patients with mitochondrial exhaustion and impaired epithelial repair	Intermediate/recovery phase	Oxygenation indices, ventilator-free days, inflammatory biomarkers	Hypoinflammatory or bioenergetically exhausted phenotypes
Glycolysis-targeted immunometabolic modulation	Hyperinflammatory glycolysis-dominant phenotypes	Early hyperinflammatory phase	Lactate kinetics, inflammatory cytokine profiles	Hyperglycolytic metabolomic signatures

Proposed conceptual framework for metabolism-informed precision trial design in ARDS integrating metabolic profiles, therapeutic timing windows, candidate translational endpoints, and potential biologic stratification approaches. The table highlights how distinct metabolic states may require different therapeutic strategies and emphasizes the importance of phenotype-informed patient selection in future translational studies

ARDS: Acute respiratory distress syndrome; MSC: Mesenchymal stromal cell; mtROS: Mitochondrial reactive oxygen species; NAD⁺: Nicotinamide adenine dinucleotide; NADH: Reduced nicotinamide adenine dinucleotide

Given the rapidly evolving landscape of metabolism-targeted interventions in ARDS, a structured overview of the current translational evidence is important to distinguish established clinical therapies from predominantly experimental or preclinical strategies. A summary of the principal metabolism-oriented therapeutic approaches discussed in this review, including their mechanistic targets, evidence levels, and current translational status, is provided in Table 5.

**Table 5:** Current translational landscape of metabolism-targeted therapeutic strategies in ARDS

Therapeutic Strategy	Proposed Mechanistic Target	Current Evidence Level	Evidence Discussed in Manuscript	Current Translational Status
Corticosteroids	Modulation of inflammatory and immunometabolic signalling pathways	High	Supported by large multicentre randomized controlled trials demonstrating clinical benefit in selected ARDS populations	Established adjunctive clinical therapy
MSC-mediated mitochondrial transfer	Restoration of mitochondrial bioenergetics, immunomodulation, and enhancement of alveolar repair	Moderate	Phase 1/2 clinical studies and recent meta-analysis demonstrating improvements in oxygenation and inflammatory markers; survival benefit remains under investigation	Early translational / investigational
Mitochondrial-targeted antioxidants (MitoQ, SS-31/elamipretide)	Reduction of mitochondrial oxidative stress (mtROS) and preservation of mitochondrial integrity	Low	Predominantly supported by preclinical ALI models demonstrating reduced oxidative injury and preservation of lung architecture	Preclinical / experimental
NAD⁺ precursor supplementation (NR, NMN)	Restoration of NAD⁺ pools and enhancement of mitochondrial respiration and sirtuin-mediated repair pathways	Low	Primarily mechanistic and preclinical evidence; no validated ARDS-specific randomized clinical trials currently available	Preclinical / experimental
Metformin-associated metabolic modulation	AMPK activation and modulation of inflammatory metabolic pathways	Low-to-moderate	Supported mainly by observational and translational critical care studies; prospective ARDS-specific randomized trial evidence remains limited	Exploratory translational stage
Itaconate-based immunometabolic modulation	Suppression of excessive inflammatory macrophage activation and enhancement of antioxidant signalling pathways	Low	Supported predominantly by mechanistic experimental studies	Preclinical / experimental
Glycolysis-targeted immunometabolic modulation	Reduction of persistent glycolytic inflammatory activation	Low	Experimental immune-cell and animal-model evidence; optimal patient selection remains uncertain	Hypothesis-generating / preclinical

Summary of metabolism-targeted therapeutic approaches discussed in the manuscript, including their proposed mechanistic targets, current evidence base, and translational status. The table highlights the distinction between therapies supported by established clinical evidence and predominantly preclinical or experimental interventions

AMPK: Adenosine monophosphate-activated protein kinase; ARDS: Acute respiratory distress syndrome; MSC: Mesenchymal stromal cell; mtROS: Mitochondrial reactive oxygen species; NAD⁺: Nicotinamide adenine dinucleotide; NMN: Nicotinamide mononucleotide; NR: Nicotinamide riboside

However, the actual clinical translational value of this framework faces a major practical hurdle: no prospective randomized clinical trial has yet successfully implemented real-time metabolic endotyping at the bedside. Real-world implementation is currently bottlenecked by the logistically prolonged turnaround times of traditional mass spectrometry or nuclear magnetic resonance platforms, which are incompatible with the rapid decision-making timelines required in the ICU [62]. Furthermore, the temporal stability of metabolic endotypes during the highly dynamic course of critical illness remains poorly defined, meaning that a patient’s metabolic profile could shift before an endotype-specific intervention takes effect [62]. Moving this strategy from a retrospective trial enrichment tool to an active bedside reality requires the development of rapid, simplified point-of-care assays targeting key surrogate intermediates [57, 62].

**Fig. 3:**
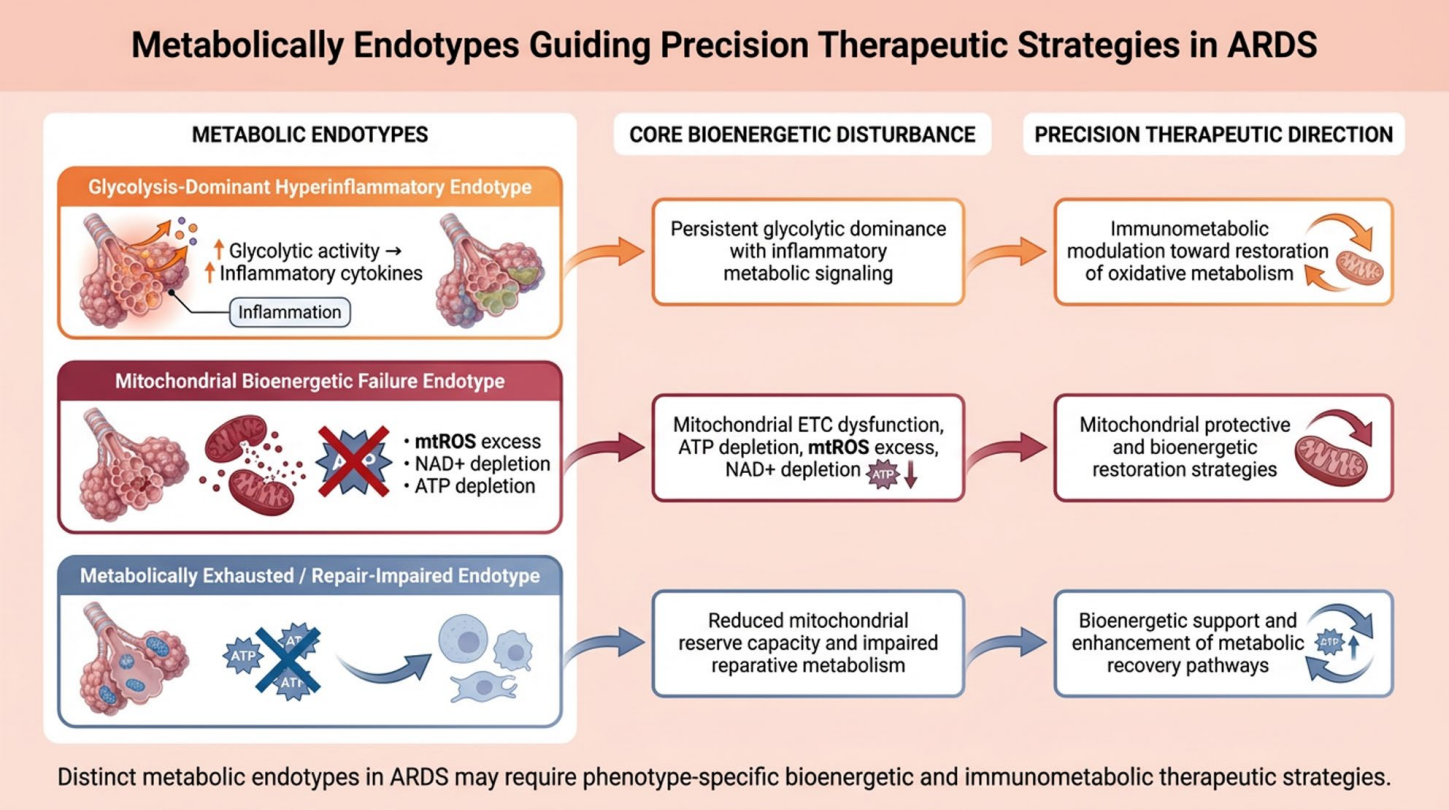
Metabolic endotypes guiding precision therapeutic strategies in ARDS. Distinct metabolic endotypes in ARDS are characterized by dominant bioenergetic disturbances that may guide targeted therapy. A glycolysis-dominant hyperinflammatory endotype suggests immunometabolic modulation, a mitochondrial bioenergetic failure endotype supports mitochondrial-protective and NAD⁺-restorative strategies, and a metabolically exhausted endotype highlights the need for bioenergetic support and recovery-focused interventions. ARDS: Acute respiratory distress syndrome; ATP: Adenosine triphosphate; ETC: Electron transport chain; mtROS: Mitochondrial reactive oxygen species; NAD⁺: Nicotinamide adenine dinucleotide; OXPHOS: Oxidative phosphorylation

Overall, therapeutic strategies aimed at restoring metabolic resilience through preservation of mitochondrial function, modulation of immunometabolic signalling, and optimization of substrate utilization may complement conventional anti-inflammatory therapies. By targeting the bioenergetic mechanisms underlying persistent lung injury, these approaches have the potential to improve recovery and reduce mortality in biologically defined ARDS subgroups, supporting the role of metabolic endotype-directed precision therapies as presented in Fig. 3 [56, 65, 66, 70, 71, 73, 75, 77].

## Future directions -toward metabolic endotyping

Future ARDS research should focus on identifying metabolic endotypes that better explain clinical heterogeneity and inform targeted therapeutic strategies. Advances in high-throughput metabolomic platforms now enable comprehensive profiling of circulating and alveolar metabolites, offering an integrated view of cellular bioenergetic status beyond traditional inflammatory biomarkers [51, 81]. These approaches may allow more precise characterization of patients based on their capacity for metabolic adaptation and recovery.

### Longitudinal metabolic monitoring

Longitudinal assessment of metabolic markers including lactate dynamics, NAD⁺-related metabolites, amino acid profiles, and acylcarnitine patterns may help identify the transition from adaptive metabolic responses to maladaptive bioenergetic failure. Monitoring these trajectories over time could reveal critical time points when targeted metabolic interventions may be most effective [18, 82]. Rather than relying on single time-point measurements, tracking metabolic recovery or deterioration over time may provide a more accurate reflection of metabolic resilience at the bedside.

### Multi-omic integration

Integrative experimental and clinical studies that simultaneously examine immunometabolism, mitochondrial function, transcriptomics, proteomics, and epithelial-endothelial barrier integrity will be essential to establish causal links between metabolic dysregulation and the severity of lung injury. Such multi-layered systems biology approaches may help identify novel therapeutic targets aimed at restoring coordinated metabolic signalling across immune and structural lung cells [83, 84]. By integrating diverse biological datasets, these strategies may also refine current phenotype classifications and identify biologically meaningful subgroups with distinct therapeutic vulnerabilities.

### Metabolism-informed clinical trials

Incorporating biological endotyping into clinical trial design is an important step toward precision medicine in ARDS. Previous studies have shown that distinct biological sub phenotypes may respond differently to therapeutic interventions, highlighting the limitations of applying uniform treatment strategies across heterogeneous patient populations [37, 56, 85]. Stratifying trial participants based on metabolic biomarkers or composite metabolomic signatures may improve trial efficiency, enhance detection of treatment effects, and reduce the likelihood of negative trials driven by biological heterogeneity [86].

Adaptive platform trials that incorporate real-time metabolic phenotyping for treatment allocation may provide a promising approach to accelerate therapeutic discovery. Such designs would allow dynamic reassignment of therapies based on evolving metabolic profiles, aligning treatment strategies with the patient’s biological state rather than relying solely on clinical severity criteria [80, 87].

### Point-of-care metabolic assays

For metabolism-informed precision therapy to be clinically feasible, rapid and deployable point-of-care assays for key metabolites are required. Current metabolomic technologies often require specialized laboratories and prolonged processing times, limiting their utility for real-time clinical decision-making. Development of bedside assays capable of measuring selected metabolic markers, combined with machine learning-based parsimonious classifiers integrating clinical and biochemical data, could enable actionable metabolic phenotyping within the critical care environment [62, 64].

### Operational limitations and standardisation challenges

Standardization of metabolomic analytical methods, harmonization of sample processing procedures, and establishment of reference standards are essential for reliable biomarker discovery and validation across studies. Variability in sample collection timing, processing protocols, and storage conditions can significantly influence metabolite measurements and contribute to inconsistent findings. Despite its compelling theoretical utility, several formidable operational hurdles currently restrict the translation of metabolic endotyping from a retrospective research tool to real-time critical care practice. First, the highly dynamic temporal course of ARDS poses a major classification challenge; host cellular metabolism undergoes rapid phase shifts across the acute injury, exudative, and fibro-proliferative stages. A static metabolic profile captured at ICU admission fails to reflect these temporal transitions, and the stability of an assigned metabolic endotype over time remains completely unverified [18, 82]. Second, a significant logistical bottleneck exists regarding platform feasibility. Gold-standard untargeted mass spectrometry and ^1^H-NMR spectroscopy require specialized infrastructure and prolonged processing turnarounds that are fundamentally incompatible with the urgent decision-making timelines required at the bedside of an unstable patient [57, 62]. Third, metabolic compartmentalization and systemic cross-talk introduce severe confounding; circulating plasma signatures often average out localized alveolar biological signals and are heavily altered by concurrent hepatic, renal, or skeletal muscle bioenergetic dysfunction common in polymorbid critical illness [10, 12]. Finally, there is a total absence of validated, prospective diagnostic cutoffs. Moving forward requires transitioning away from descriptive, bulk-averaged high-throughput discovery datasets toward simple, parsimonious biomarker panels validated across geographically diverse, prospective clinical trial networks [57, 85]. Multicentre collaborative studies using standardized protocols will therefore be necessary to support the reliable translation of metabolic biomarkers into clinical practice [62, 63].

Overall, progress toward metabolic endotyping will require coordinated integration of longitudinal metabolomic analyses, multi-omic systems biology approaches, and precision-oriented clinical trial design. Such efforts may help identify biologically defined patient subgroups with distinct metabolic vulnerabilities, thereby enabling targeted interventions aimed at restoring metabolic resilience and improving outcomes in ARDS [63, 64, 81, 82, 84, 86, 87].

## Conclusion

ARDS remains a biologically heterogeneous syndrome in which patients exposed to similar initial insults often follow markedly different clinical trajectories. Although inflammation is central to its pathogenesis, inflammatory burden alone does not fully explain differences in disease progression, treatment response, or outcomes. Evidence reviewed here suggests that variation in metabolic resilience which is the ability of cells to restore mitochondrial oxidative metabolism, maintain redox balance, and regulate metabolite signalling following inflammatory stress, may be a key factor underlying this heterogeneity.

Inflammatory injury in ARDS is accompanied by immunometabolic reprogramming, mitochondrial dysfunction, and accumulation of bioactive metabolites across immune, epithelial, and endothelial compartments. When metabolic balance is restored, inflammatory signalling declines and tissue repair mechanisms are activated. In contrast, failure to re-establish coordinated bioenergetic control leads to persistent glycolytic metabolism, oxidative stress, sustained inflammasome activation, and progressive barrier dysfunction, ultimately contributing to refractory hypoxemia and organ failure. This framework helps explain the emergence of hyperinflammatory and hypo inflammatory ARDS phenotypes and the variable responses to current therapies. Rather than representing distinct disease entities, these phenotypes may reflect different positions along a continuum ranging from preserved metabolic resilience to progressive metabolic failure [88].

Recognizing metabolic resilience as a key determinant of ARDS heterogeneity has important translational implications. Longitudinal assessment of metabolic markers and metabolomic profiling may enable early identification of patients with impaired bioenergetic adaptation who are at increased risk of persistent lung injury and poor outcomes. Therapeutic strategies aimed at preserving mitochondrial function, restoring NAD⁺ homeostasis, and modulating maladaptive metabolite signalling may therefore complement existing supportive care and support more individualized treatment approaches.

Overall, conceptualizing ARDS through the framework of metabolic resilience integrates immunometabolic reprogramming, mitochondrial dysfunction, and metabolite-driven signalling into a unified biological model. This perspective links cellular bioenergetics with phenotype divergence, clinical trajectory, and treatment responsiveness, and provides a foundation for future metabolism-informed endotyping and precision therapeutic strategies in ARDS.

## Data Availability

Not applicable.

## Abbreviations

AMPK: Adenosine Monophosphate-Activated Protein Kinase
ARDS: Acute Respiratory Distress Syndrome
ATP: Adenosine Triphosphate
BALF: Bronchoalveolar Lavage Fluid
COPD: Chronic Obstructive Pulmonary Disease
ETC: Electron Transport Chain
GRα: Glucocorticoid Receptor Alpha
GRADE: Grading of Recommendations Assessment, Development and Evaluation
HIF-1α: Hypoxia-Inducible Factor-1α
ICU: Intensive Care Unit
IL-1β: Interleukin-1β
IL-18: Interleukin-18
LC-MS: Liquid Chromatography-Mass Spectrometry
MitoQ: Mitoquinone
MSC: Mesenchymal Stromal Cell
mtDAMPs: Mitochondrial Damage-Associated Molecular Patterns
mtDNA: Mitochondrial Deoxyribonucleic Acid
mtROS: Mitochondrial Reactive Oxygen Species
NAD⁺: Nicotinamide Adenine Dinucleotide
NADH: Reduced-Nicotinamide Adenine Dinucleotide
NET: Neutrophil Extracellular Trap
NF-κB: Nuclear Factor-κB
NLRP3: NOD-,LRR- and Pyrin Domain-Containing Protein 3
NR: Nicotinamide Riboside
NMN: Nicotinamide Mononucleotide
Nrf2: Nuclear Factor Erythroid 2-Related Factor 2
Na⁺/K⁺-ATPase: Sodium-Potassium ATPase
PINK1: PTEN-induced kinase 1
RCT: Randomized Controlled Trial
RhoA-ROCK: RhoA-Rho-Associated Coiled-Coil Containing Protein Kinase
ROS: Reactive Oxygen Species
TCA: Tricarboxylic Acid
TLR4: Toll-Like Receptor 4
